# Three-Dimensional Analysis of a Viral RNA Replication Complex Reveals a Virus-Induced Mini-Organelle

**DOI:** 10.1371/journal.pbio.0050220

**Published:** 2007-08-14

**Authors:** Benjamin G Kopek, Guy Perkins, David J Miller, Mark H Ellisman, Paul Ahlquist

**Affiliations:** 1 Institute for Molecular Virology, University of Wisconsin–Madison, Madison, Wisconsin, United States of America; 2 National Center for Microscopy and Imaging Research, University of California San Diego, La Jolla, California, United States of America; 3 Department of Neurosciences, University of California San Diego, La Jolla, California, United States of America; 4 Department of Medicine, University of Michigan Medical School, Ann Arbor, Michigan, United States of America; 5 Department of Microbiology and Immunology, University of Michigan Medical School, Ann Arbor, Michigan, United States of America; 6 Howard Hughes Medical Institute, University of Wisconsin–Madison, Madison, Wisconsin, United States of America; Washington University School of Medicine, United States of America

## Abstract

Positive-strand RNA viruses are the largest genetic class of viruses and include many serious human pathogens. All positive-strand RNA viruses replicate their genomes in association with intracellular membrane rearrangements such as single- or double-membrane vesicles. However, the exact sites of RNA synthesis and crucial topological relationships between relevant membranes, vesicle interiors, surrounding lumens, and cytoplasm generally are poorly defined. We applied electron microscope tomography and complementary approaches to flock house virus (FHV)–infected *Drosophila* cells to provide the first 3-D analysis of such replication complexes. The sole FHV RNA replication factor, protein A, and FHV-specific 5-bromouridine 5'-triphosphate incorporation localized between inner and outer mitochondrial membranes inside ∼50-nm vesicles (spherules), which thus are FHV-induced compartments for viral RNA synthesis. All such FHV spherules were outer mitochondrial membrane invaginations with interiors connected to the cytoplasm by a necked channel of ∼10-nm diameter, which is sufficient for ribonucleotide import and product RNA export. Tomographic, biochemical, and other results imply that FHV spherules contain, on average, three RNA replication intermediates and an interior shell of ∼100 membrane-spanning, self-interacting protein As. The results identify spherules as the site of protein A and nascent RNA accumulation and define spherule topology, dimensions, and stoichiometry to reveal the nature and many details of the organization and function of the FHV RNA replication complex. The resulting insights appear relevant to many other positive-strand RNA viruses and support recently proposed structural and likely evolutionary parallels with retrovirus and double-stranded RNA virus virions.

## Author Summary

Whereas cells store and replicate their genomes as DNA, most viruses have RNA genomes that replicate by using virus-specific pathways in the host cell. The largest class of RNA viruses, the positive-strand RNA viruses, replicate their genomes on intracellular membranes. However, little is understood about how and why these viruses use membranes in RNA replication. The well-studied flock house virus (FHV) replicates its RNA on mitochondrial membranes. We found that the single FHV RNA replication factor and newly synthesized FHV RNA localized predominantly in numerous infection-specific membrane vesicles inside the outer mitochondrial membrane. We used electron microscope tomography to image these membranes in three dimensions and found that the interior of each vesicle was connected to the cytoplasm by a single necked channel large enough to import ribonucleotide substrates and to export product RNA. The results suggest that FHV uses these vesicles as replication compartments, which may also protect replicating RNA from competing processes and host defenses. These findings complement results from other viruses to support possible parallels between genome replication by positive-strand RNA viruses and two distinct virus classes, double-stranded RNA and reverse-transcribing viruses.

## Abstract 3

All positive-strand RNA viruses replicate their genomes in association with intracellular membrane rearrangements; here, a three-dimensional analysis of these complexes in flock house nodavirus-infected *Drosophila* cells provides insight into the replication process.

## Introduction

Positive-strand RNA [(+)RNA] viruses contain messenger-sense, single-stranded RNA in their virions; they represent over a third of known virus genera; and they include many important human, animal, and plant pathogens [1]. A common, if not universal, feature of (+)RNA virus replication is the association of their RNA replication complexes with infection-specific host intracellular membrane rearrangements [2–19]. Characterizing the features of these membrane-associated RNA replication complexes should identify general principles and mechanisms of (+)RNA virus replication and could lead to broadly applicable control strategies for (+)RNA viruses including, e.g., hepatitis C virus and the SARS coronavirus.

For many (+)RNA viruses—including alphaviruses [5], other members of the alphavirus-like superfamily [15], rubiviruses [7,20], flaviviruses [21], tombusviruses [22], and others [4,23–25] —RNA replication occurs in association with ∼50–70-nm diameter membranous vesicles or spherules that form in the lumen of specific secretory compartments or organelles. The similarity of these structures suggests that RNA replication by such otherwise distinct viruses involves important conserved features related to membranes. For some viruses, the localization of viral replicase proteins [11,17,23,26–28] or viral RNA synthesis [5,15,29] suggest that such spherules may contain or comprise the viral RNA replication complex. For brome mosaic virus (BMV) and some other viruses, two-dimensional (2-D) electron microscopy (EM) reveals that a fraction of such spherules have interiors that appear to be connected to the cytoplasm by membranous necks [15,25,28]. However, limitations inherent in random sectioning and 2-D analysis prevent standard EM from resolving many issues crucial to understanding spherule structure and function, such as the range of spherule diameter and volume, and whether all spherule interiors are connected to the cytoplasm or if some bud free from their adjacent bounding membranes.

To resolve these and other issues central to the mechanism of RNA replication, we used EM tomography (EMT) to provide the first, to our knowledge, three-dimensional (3-D) ultrastructural study of the membrane-bound RNA replication complexes of a (+)RNA virus. EMT generates high-resolution, 3-D images or tomograms by digitally processing a series of 50–100 electron micrographs collected as a specimen is tilted in 1–2 ° increments on an axis perpendicular to the electron beam [30]. Similar 3-D EMT analyses have been crucial to reveal many important features of complex cellular organelles such as the Golgi apparatus [31–34], endoplasmic reticulum [33,34], and mitochondria [35–37].

We chose flock house virus (FHV), the best characterized member of the *Nodaviridae,* as a (+)RNA virus with advantageous features for such studies. FHV has been used as a model to study RNA replication [8,9,38–40], virion structure and assembly [41,42], and genomic packaging [42–46]. FHV has a 4.5-kb bipartite RNA genome in which RNA2 (1.4 kb) encodes the capsid precursor [47] whereas RNA1 (3.1 kb) encodes an RNA silencing inhibitor [48,49] and a multifunctional RNA replication factor, protein A [40,50,51]. Protein A, the only FHV protein needed for RNA replication, is directed by an N-terminal targeting and transmembrane sequence to outer mitochondrial membranes, where it colocalizes by immunofluorescence with the sites of viral RNA synthesis [8,38]. Gradient flotation and dissociation assays showed that protein A behaves as an integral transmembrane protein [38]. Additionally, protease digestion and selective permeabilization after differential epitope tagging demonstrated that protein A is inserted into the outer mitochondrial membrane with the N terminus in the inner membrane space or matrix, while the majority of the protein A sequence is exposed to the cytoplasm [38]. Protein A also self-interacts in vivo in ways that are important for RNA replication [52]. Like many other (+)RNA viruses [4,5,7,15,20–25], FHV infection induces the formation of ∼50-nm membranous vesicles or spherules, which, for the case of FHV, are found between the mitochondrial outer and inner membranes [8].

Here we use EMT and multiple complementary approaches to provide 3-D visualization of a (+)RNA virus replication complex. Among other findings, the results show that FHV spherules are compartments or mini-organelles for viral RNA synthesis, which form by invagination of the outer mitochondrial membrane and communicate with the cytoplasm through ∼10-nm diameter necks. The results further indicate that each spherule contains, on average, ∼100 membrane-spanning, self-interacting protein A molecules and that FHV-infected cells contain 2–4 genomic RNA replication intermediates per spherule. These observations define a new level of understanding of the nature, structure, and organization of a viral RNA replication complex, including principles that are likely relevant to many other (+)RNA viruses.

## Results

### FHV RNA Replication Protein A and RNA Synthesis Are Located within Membrane-Bound Spherules

**Figure 1:**
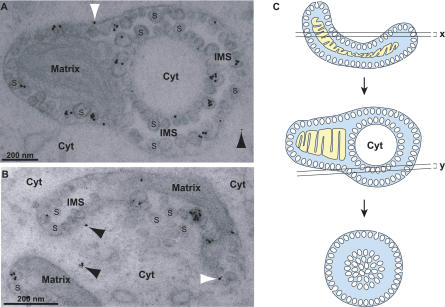
FHV Protein A Is Localized in Virus-Induced Mitochondrial Spherules (A and B) Examples of anti-protein A immunogold labeling of mitochondrial spherules in FHV-infected cells. Cells were fixed in 4% paraformaldehyde and 0.2% glutaraldehyde, postfixed in 0.1% osmium, dehydrated and embedded in LR Gold resin, sectioned and placed on nickel-grids, immunostained with protein A antisera and a secondary antibody conjugated to ultrasmall gold particles, and silver-enhanced. The mitochondrial matrix, cytoplasm (Cyt) and examples of the many spherules (S) in the mitochondrial intermembrane space (IMS) are labeled for reference. White arrowheads indicate gold particles at a mitochondrial membrane but not directly over a spherule. Black arrowheads indicate gold particles in the cytoplasm. As illustrated in panel A, islands of cytoplasm surrounded by a mitochondrial ring are seen frequently in EM sections of FHV-infected mitochondria. (C) A schematic, based on 3-D tomographic analysis shown below, of how such cytoplasmic islands are generated by EM sectioning of the frequently cup-shaped, FHV-modified mitochondria [see panel (B)]. The bottom image in (C) further shows how some planes of sectioning give rise to “vesicle packet” structures seen in Figures 2C and 3D below. White, cytoplasm; blue, space between outer and inner mitochondrial membranes; yellow, matrix.

Protein A is the only FHV protein needed for RNA replication and so must co-localize with viral RNA replication complexes. Prior immunofluorescence and immunogold labeling EM localized protein A to the outer mitochondrial membrane in FHV-infected cells [8]. However, in those prior attempts at immunogold labeling, fixation conditions needed to preserve spherule ultrastructure abolished protein A antigenicity for the polyclonal antibody used, hence blocking protein A localization relative to spherules. To overcome this, we identified a monoclonal antibody against protein A [9] that was able to detect protein A under fixation conditions that sufficiently retained spherule ultrastructure. Immunogold EM with this protein A monoclonal antibody revealed that nearly all protein A was in or on mitochondrial spherules in FHV-infected cells (Figure 1). Over 900 gold particles in 25 different electron micrographs were counted and 88% ± 5% of the specific gold labeling density above background (see Materials and Methods) was associated with spherules. Cytoplasmic labeling, presumably including protein A being translated and/or trafficked in the cytoplasm, was just 2% ± 7% above background labeling levels. The remaining 10% ± 5% of immunogold label was associated with mitochondria but not discernable spherules, including gold particles on the cytoplasmic face of the outer mitochondrial membrane where some protein A might have been localized that was not, or not yet, internalized into spherules. The clustering pattern of immunogold particles in a subset of spherules may be due to nonuniform epitope exposure or signal amplification by secondary antibodies. To avoid over-weighting the calculations due to such clustering effects, we also analyzed the same 25 micrographs counting each cluster as one event. The resulting count of clusters gave a very similar pattern to the one described above (94% spherule associated).

**Figure 2:**
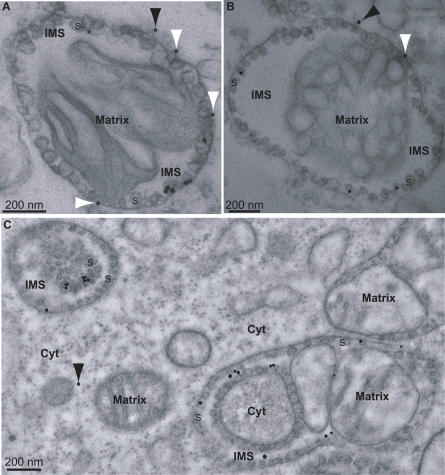
Association of Viral RNA Synthesis with FHV Spherules (A and B) Examples of incorporated BrUTP immunogold labeling of mitochondrial spherules from cells infected with FHV. Isolated mitochondria were incubated for 1 h at 28 °C with a transcription mix that included BrUTP. (C) Localization of RNA synthesis to spherules in FHV-infected cells into which BrUTP was introduced by transfection. Length of labeling period was 15 min. Samples were prepared for EM and immunogold labeling as in Figure 1, except that anti-BrU antibody was used instead of anti-protein A. See Figure 1C for an explanation of the nonstandard mitochondrial morphologies seen in the upper left and lower right of (C). The arrowheads and labeling of mitochondria, spherules, and cytoplasm are as in Figure 1. In interpreting the immunogold localization, note that the primary-secondary antibody complex linking the gold particles to their target epitopes may span up to 20 nm.

**Figure 3:**
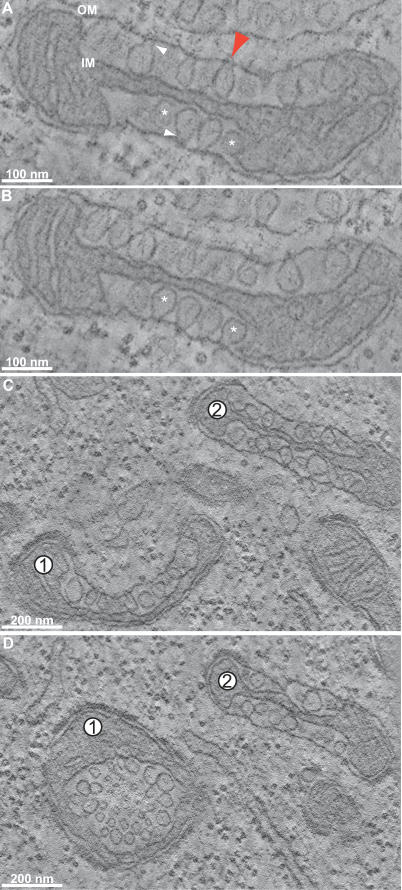
EMT Reconstructions of Mitochondria from FHV-Infected *Drosophila* Cells Labels denote outer mitochondrial membrane (OM) and inner mitochondrial membrane (IM). (A and B) Slices through a tomographic reconstruction showing FHV-induced spherule rearrangements of a mitochondrion (see Video S1 to view the entire *z*-series). (B) is a slice that is ∼15 nm down the *z*-axis (perpendicular to the plane of the image) from the slice in (A). White arrowheads indicate the necks that connect spherules to the OM. Asterisks mark two spherules that appear to be free vesicles in (A) but are shown to have necked connections to the outer membrane in (B). A red arrow marks the ∼10-nm channel connecting a representative spherule interior to the cytoplasm. This red arrow also corresponds to the same spherule and connection as the red arrow in Figure 4B–4C. (C and D) Images from another tomogram that are displaced from each other in the *z*-axis by ∼150 nm. Note the change in morphology of mitochondrion 1 where spherules that appear to be connected to the outer mitochondrial membrane in (C) appear as a vesicle packet in (D).

**Figure 4:**
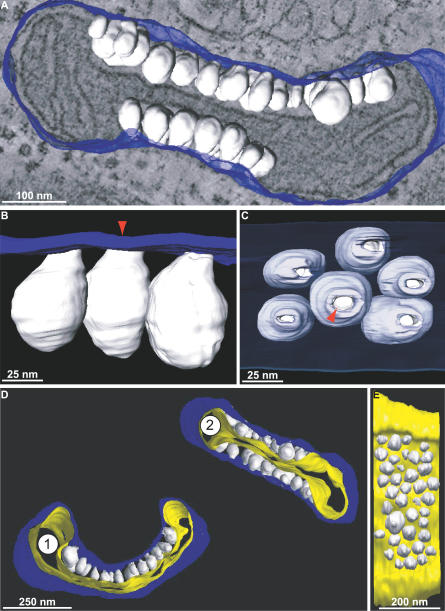
3-D Maps of FHV-Modified Mitochondria Blue indicates outer mitochondrial membrane, white indicates FHV spherules, yellow indicates inner mitochondrial membrane. (A) Merged image of a 3-D map of the outer membrane and spherules of the mitochondrion from Figure 3A and 3B and a slice of the tomogram showing the electron density map from which it was derived. (B) A portion of the map in (A) showing a close-up view of the connections between the outer mitochondrial membrane and the spherules. The red arrow marks that same spherule as the one depicted by the red arrow in Figure 3A. (C) A 90 ° rotation of (B) showing the channels that connect the spherule interiors to the cytoplasm. The outer membrane has been made translucent to show the spherules behind it. Again, the red arrow corresponds to the red arrows in Figures 3A and 4B. (D) 3-D maps of the mitochondria shown in Figure 3C. (E) A view of mitochondrion 2 from (D) that has been rotated and has had the outer membrane removed to show the range of spherule sizes.

By immunofluorescence microscopy, we found that 5-bromouridine (BrU)-labeled FHV RNA synthesis occurs exclusively at outer mitochondrial membranes in infected *Drosophila* cells [8]. To localize more precisely FHV RNA synthesis in relation to spherules, we incubated mitochondria isolated from uninfected and FHV-infected *Drosophila* cells with a nucleotide mix including 5-bromouridine 5'-triphosphate (BrUTP) and performed immunogold labeling EM with an antibody recognizing BrU incorporated into RNA, but not unincorporated BrUTP.

For mitochondrial preparations from FHV-infected cells, spherules were the major site of immunogold labeling (Figure 2A and 2B). Of 221 gold particles examined, 70% ± 18% were on spherules. The remaining 30% of gold particles that fell outside of spherules may include mature RNA products released from spherules and nonspecific background labeling. For mitochondria from uninfected cells, background labeling levels were independent of the addition or omission of BrUTP and averaged 15% of the total immunogold labeling of BrUTP-treated mitochondria from FHV-infected cells. We found that using isolated mitochondria was advantageous for the BrUTP-labeling experiments because of low transfection efficiencies of BrUTP into whole *Drosophila* cells. Nevertheless, we were able to obtain some immunogold labeling results using intact *Drosophila* cells, which also showed that spherules were the major sites of BrUTP-labeling (Figure 2C). Gold particles in the intermembrane space of the mitochondrion in the lower right are well within the distance (20 nm) from spherules that may be spanned by the primary and secondary antibodies linking the immunogold particles to their target epitopes [53].

### All Replication Spherules Retain an Open Connection to the Cytoplasm

Having shown that spherules were the sites of protein A accumulation and FHV RNA synthesis, we applied 3-D EMT to provide a new level of analysis of spherule morphology and topology. As noted in the Introduction, the 3-D nature of EMT overcomes many serious limitations of 2-D EM analysis to reveal possible connections to surrounding membranes and compartments, complete dimensions, and other fundamental characteristics not accessible from conventional transmission EM analyses of random sections. For example, along the *z*-axis parallel to the electron beam, standard transmission EM projects a 50–70-nm section into a single view, whereas EMT allows computationally dissecting an entire ∼250-nm-thick sample volume into successively viewable planes spaced with a resolution of just a few nanometers [54].

To produce 3-D reconstructions of FHV-infected cells including modified mitochondria, *Drosophila* S2 cells were harvested 12 h post infection (hpi) and fixed, embedded, and sectioned as described under Materials and Methods. For each reconstruction, a tilt series of 60 images was collected by rotating a 250-nm-thick section of resin-embedded sample in 2 ° increments between −60 ° to +60 ° relative to the plane perpendicular to the beam, and was digitally processed to produce a tomographic reconstruction. Using *Drosophila* cells from three independent FHV infection experiments, five independent reconstructions were generated using a single-tilt series technique (Figure 3C–3D and additional unpublished data) and one reconstruction was performed using a double tilt technique (Figure 3A and 3B) to improve tomographic resolution further [55]. Representative results are shown in the figures.

For one such tomogram, Figure 3A shows the image of a computationally dissected, 2.2-nm-thick virtual section, revealing an FHV-modified mitochondrion containing spherules in the mitochondrial intermembrane space. This 2-D image shows a typical view of randomly sectioned, FHV-modified mitochondria, in which some spherules appear to be light bulb–shaped invaginations attached to the outer membrane by small diameter necks (white arrowheads), whereas others appear to be free vesicles in the intermembrane space (asterisks). Figure 3B shows another virtual section from the same tomogram, displaced down the perpendicular *z*-axis by ∼15 nm to a point where those spherules that appeared to be free vesicles in Figure 3A (asterisks) now show necked attachments to the outer membrane. To determine if all spherules were attached to the outer mitochondrial membrane, or if a population of spherules budded free of this membrane, we followed individual spherules through dozens of successive 2.2-nm-spaced adjacent planes perpendicular to the electron beam (a “*z*-series” of sections). When all six reconstructions were examined in this way, all ∼500 spherules in all ∼8 FHV-modified mitochondria examined were found to be connected to the outer mitochondrial membrane by a membranous neck observable in some plane of the sample. The red arrowhead in Figure 3A points to a channel through the spherule neck that connects the interior of a spherule to the cytoplasm.

Thus, all spherules are necked invaginations of the outer mitochondrial membrane whose interiors remain connected to the cytoplasm, and sections in which a given spherule appears to be a free vesicle simply represent planes that did not pass through the smaller diameter neck linking the spherule membrane to the mitochondrial outer membrane. This is illustrated more dynamically in Video S1, which animates the progression through a *z*-series of sections of the tomogram of Figure 3A and 3B.

Figure 3C–3D shows two virtual sections from another tomogram, which are displaced ∼150 nm down the perpendicular *z*-axis from each other. As shown in a video through this *z*-series (Video S2), mitochondrion 1 curves significantly in the space between these two sections, such that the plane of Figure 3C sections mitochondrion 1 spherules parallel to an axis through the spherule necks, whereas the parallel plane of Figure 3D sections the spherules on another part of the mitochondrion 1 surface tangential to the axes through their necks. These two perpendicular views of similar spherules on the same mitochondrion are notable because Figure 3C strongly resembles images of spherules induced by alphaviruses, nodaviruses, etc. [8,28], whereas Figure 3D resembles images of apparently distinct “vesicle packets” described for flaviviruses [3]. Thus, some apparently distinct membrane rearrangements and vesicle structures observed in connection with RNA replication by different (+)RNA viruses may represent related structures distinguished in part by the perspective from which they were viewed.

### 3-D Mapping of Spherule Membranes

To generate 3-D surface maps of the virus-induced membrane rearrangements associated with FHV RNA replication, we manually traced the inner and outer mitochondrial membranes (including spherules) over ∼100 adjacent, 2.2-nm-spaced virtual sections of selected tomographic reconstructions, and we used a computer-generated mesh overlay to join these tracings into continuous surfaces (Figure 4). Figure 4A shows part of the relationship between the electron density of the mitochondrion in Figure 3A and its 3-D map, and Video S3 provides a much more dynamic visualization of this relationship and the complete 3-D map. For clarity, the cytoplasmic faces of outer mitochondrial membranes are colored blue, spherule membranes are white, and inner mitochondrial membranes are yellow.

**Figure 5:**
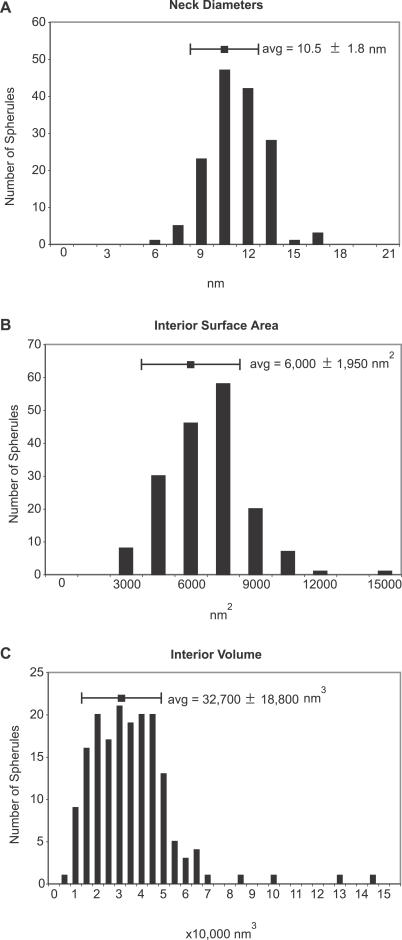
Spherule Dimensions Distribution of spherule neck channel diameters (A), interior surface areas (B), and interior volumes (C). The data shown represents measurements of 150 (A) and 175 (B and C) individual spherules.

Figure 4B shows a close-up view of a portion of the 3-D map in Figure 4A that demonstrates the connection of the spherules to the outer mitochondrial membrane. This and other similar maps confirmed as noted above that the spherule membranes (white) are continuous with the outer mitochondrial membrane (blue). Figure 4C is a 90 ° rotation of Figure 4B that shows a view looking down on the surface of an FHV-modified mitochondrion, with the outer membrane (blue) rendered translucent to reveal the spherules beneath (Video S4). The necked channels connecting the interior of each spherule to the cytoplasm (red arrowhead) are clearly visible as circular openings in the outer membrane. For 150 individual spherules in four mitochondria from four cells and three experiments, we measured the interior diameters of these neck channels as the distance between the two lipid bilayers, from inner leaflet to inner leaflet, at the point where the tomographic plane sliced through the center of the neck. The resulting distribution of neck diameters is shown in Figure 5A. The average diameter of the neck channel was 10.5 ± 1.8 nm (Figure 5A), which is more than large enough to allow import of ribonucleotides and export of RNA products (diameter < 2 nm).

Surface-rendered, 3-D maps of the two mitochondria from Figure 3C are shown in Figure 4D, illustrating also the inner mitochondrial membrane (yellow). Using such surface-rendered maps (Figure 4 and other unpublished data), we also measured the interior volume and membrane surface area of 175 spherules. As illustrated in Figure 5C, the spherule volumes spanned a range of ∼15,000 to 50,000 nm^3^. A range of spherule sizes is seen in Figure 4E, which is a rotated view of mitochondrion 2 in Figure 4D, with the outer membrane removed. The average spherule interior volume was ∼33,000 nm^3^ (Figure 5C), and the average interior spherule membrane surface area was ∼6,000 nm^2^ (Figure 5B).

### Stoichiometry of FHV RNA Replication Components Reveals High Protein A Copy Number

**Figure 6:**
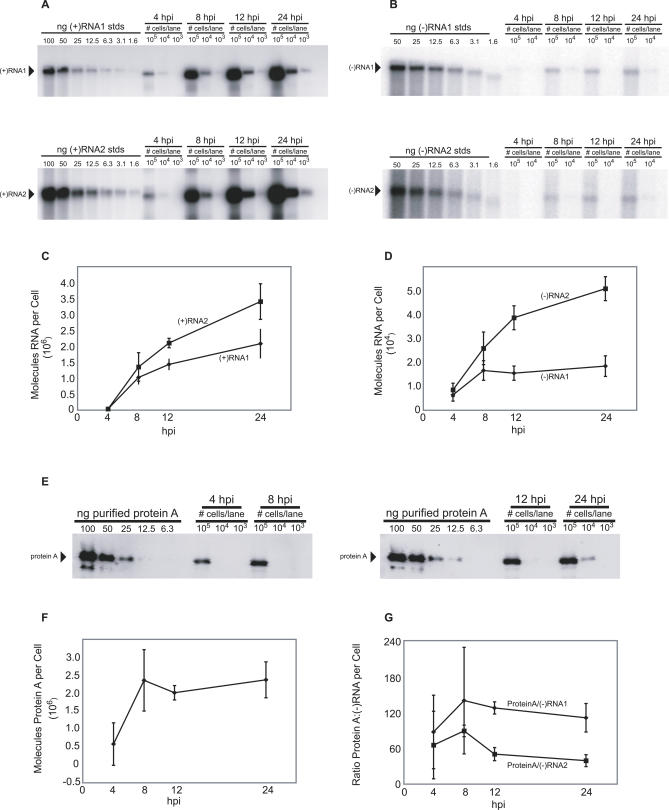
Measurements of the number of molecules of FHV (+)RNAs and (−)RNAs and protein A per cell in FHV-infected *Drosophila* cells (A and B) Cells were harvested and counted at the time points indicated above the figure, and total RNA was extracted. An amount of RNA corresponding to 1.0 × 10^5^, 1.0 × 10^4^, or 1.0 × 10^3^ cell equivalents was loaded as indicated above each lane and subjected to Northern blot hybridization with radio-labeled probes specific for the detection of (+)RNA1 (top panel of A), (−)RNA1 (top panel of B), (+)RNA2 (bottom panel of A), or (−)RNA2 (bottom panel of B). Specific signals are indicated by the arrowheads. (C) Graph of the number of molecules of (+)RNA1 (diamond) and (+)RNA2 (squares) per cell over a 24-h time course. (D) Graph of the number of molecules of (−)RNA1 and (−)RNA2 per cell over a 24-h time course. (E) An aliquot of the same cells harvested for the RNA analysis at the indicated time points was lysed in Laemmli sample buffer and subjected to immunoblot analysis with polyclonal antisera for protein A. Protein A levels were measured by comparison with the signal intensities derived from known amounts of purified protein A. (F) Graph of the number of molecules of protein A per cell over a 24-h time course. (G) Graph of the ratios of protein A versus (−)RNA1 (diamonds) and (−)RNA2 during the course of infection. The 24 hpi data shown represent the mean and standard deviation for three independent experiments. The data shown for 4, 8, and 12 hpi represent the mean and standard deviation for two independent experiments.

Since both protein A (Figure 1) and nascent FHV RNA (Figure 2) localized predominantly or exclusively to spherules, the relative numbers of protein A, RNA replication templates, and spherules could provide important insights into the structure and organization of FHV RNA replication complexes. Accordingly, we measured the number of molecules of protein A and FHV RNAs per cell in *Drosophila* S2 cells at 4, 8, 12, and 24 hpi with FHV. The numbers of positive- and negative-strand genomic RNAs per cell were measured by quantitative Northern blotting calibrated with known amounts of in vitro transcripts (Figure 6A and 6B). The number of protein A molecules per cell was measured by quantitative Western blotting calibrated with known amounts of co-electrophoresed, purified protein A standards (Figure 6E).

Starting before 4 hpi and continuing thereafter in FHV-infected *Drosophila* cells, the primary mode of viral RNA synthesis is (+)RNA synthesis from negative-strand RNA [(−)RNA] templates (Figure 6A–6D). The number of (+)RNA1 and (+)RNA2 per cell increased from ∼40,000 molecules of each RNA species at 4 hpi to ∼2–3 million each by 24 hpi (Figure 6C). Such (+)RNA products primarily accumulate in the cytoplasm for translation and encapsidation, and only a minor fraction of (+)RNAs fractionate with the membrane-associated RNA replication complex (P. Van Wynsberghe, P. Ahlquist, unpublished data).

By contrast to positive-strand export and accumulation in the cytoplasm, FHV (−)RNAs appear to function only as RNA replication intermediates and are completely membrane-associated (P. Van Wynsberghe, P. Ahlquist, unpublished data). (−)RNA thus is a key measure of a minimal RNA replication complex, because every mature RNA replication complex, active in (+)RNA synthesis, must contain at least one (−)RNA template. Therefore, the number of (−)RNAs gives an estimate of the maximal number of replication complexes per cell. (−)RNA1 accumulation plateaued by 8 hpi at ∼16,000 copies per cell (Figure 6D). (−)RNA2 accumulation increased throughout the first 24 hpi, although more slowly after 12 hpi, reaching ∼50,000 molecules per cell by 24 hpi (Figure 6D).

The number of protein A molecules plateaued by 8 hpi (Figure 6F), which is consistent with prior results that protein A synthesis occurs early in infection and then declines [47]. Intriguingly, the peak level of protein A was ∼2 million molecules per cell (Figure 6F). Protein A was thus present at dramatically higher levels than (−) RNA templates were. The ratio of protein A to (−)RNAs was relatively consistent over all time points examined, with averages throughout infection of 118 ± 23 and 64 ± 20 protein A copies per (−)RNA1 and (−)RNA2, respectively (Figure 6G).

To understand the organization of the replication complex in relation to the spherules better, we compared the number of spherules per cell with the number of protein A and (−)RNA molecules per cell. To measure the number of spherules per cell, we collected FHV-infected *Drosophila* S2 cells at 12 hpi, processed them for transmission EM, and imaged 25 randomly sectioned cell profiles. All spherules in each imaged cell section were counted and divided by the cell section volume, which was calculated by measuring the cell area using ImageJ (National Institutes of Health) and multiplying by the effective section thickness (see Materials and Methods). The number of spherules per cell was calculated by multiplying the resulting density of spherules by the average volume of the almost perfectly round, 10 μm-diameter *Drosophila* S2 cells [56] (and our independent, matching measurements).

These calculations revealed the average number of spherules per cell at 12 hpi to be ∼20,000 ± 11,000 (Table 1). The ratio of protein A per cell to spherules per cell revealed that on average, there are ∼100 copies of protein A per spherule (Table 1). Further comparison to the Figure 6 data shows that, on average there are ∼1 (−)RNA1 and ∼2 (−)RNA2 molecules per spherule (Table 1). The implications of these results for the organization of replication complexes are considered further in the Discussion.

**Table 1:**
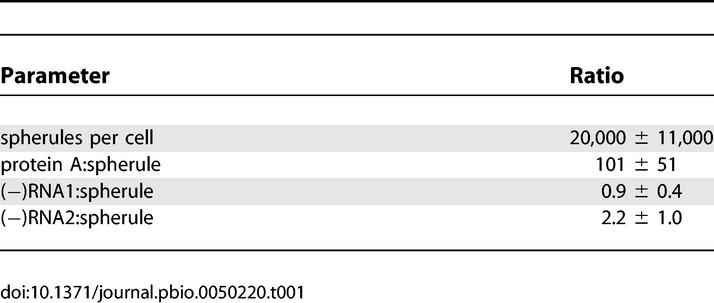
Ratio of Spherules per Cell to Protein A and (−)RNA at 12 hpi

## Discussion

To advance understanding of the crucial relationship between (+)RNA viruses and the intracellular membranes on which they replicate their RNA genomes, we combined 3-D ultrastructural imaging with quantitative biochemical data and other results to model the architecture and organization of a nodavirus RNA replication complex. Immunogold labeling identified virus-induced membranous spherules as the sites of accumulation of the sole FHV RNA replication protein, protein A, and of FHV RNA synthesis (Figures 1 and 2). EMT revealed that all FHV spherules maintain an open connection with the cytoplasm with a diameter of ∼10 nm, which is wide enough to allow the exchange of ribonucleotides and RNA products (Figures 4C and 5A). Our stoichiometry measurements further revealed the presence of, on average, 100 copies of the viral replicase protein A and 2–4 RNA replication intermediates per spherule (Table 1). As discussed further below, these findings have substantial implications for the structure, assembly, and function of the FHV RNA replication complex and likely also for the organization of many similar membrane-associated viral RNA replication complexes. In addition to advancing understanding of viral replication mechanisms, such insights also should prove valuable for developing additional antiviral strategies or agents.

### Organization of the FHV RNA Replication Complex

**Figure 7:**
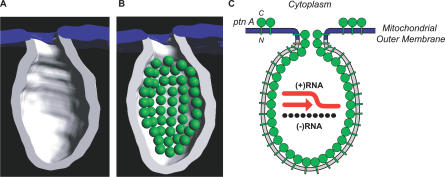
Modeling of FHV Transmembrane Protein A into a Spherule (A) 3-D map of a single spherule where half of the membrane has been removed to show the interior. As in Figure 4, the spherule membrane is white and the contiguous outer mitochondrial membrane is blue. (B) Schematic of likely protein A organization within a spherule. Based on the average density of globular proteins, FHV protein A (112 kDa) is modeled in as a green sphere of ∼7 nm in diameter. Based on the average of ∼100 protein A molecules per spherule (Table 1), the figure shows the potential packing arrangement of 50 protein A molecules in half a spherule. The spheres are shown lining the interior surface area of the spherule membrane because protein A is a transmembrane protein [38]. (C) Schematic representation of the structure and organization of the FHV RNA replication complex. Protein A (*ptn A*; green spheres) forms a shell within the mitochondrial membrane spherule within which RNA synthesis occurs (*N* = N terminus; *C* = C terminus). Protein A is also shown as possibly extending into the spherule neck, since it may be a determinant of the relatively constant 10-nm diameter neck. As noted in Figure 1 (white arrowheads), a small fraction of protein A may reside on the outer mitochondrial membrane external to spherules. The diagram shows (+)RNA synthesis (red arrow) from (−)RNA templates (black segmented line), which is the predominant form of FHV RNA synthesis throughout all but the earliest phases of FHV infection (Figure 6A–6D).

Protein A is a transmembrane protein in outer mitochondrial membranes [38] and is ∼90% localized within spherules (Figure 1). Therefore, protein A must line the interior membrane surface of spherules. If protein A is similar to typical globular proteins, its volume would be ∼183 nm^3^, based on the protein A molecular weight of 112 kDa [57] and the average partial specific volume of typical proteins [58]. If globular, protein A then would have a diameter of ∼7 nm and cover a surface area of ∼40 nm^2^. Thus, the average spherule interior membrane surface area of 6,000 nm^2^ (Figure 5B) provides enough space to accommodate at most ∼150 protein A molecules, under a perfect close-packing arrangement. Therefore, the measured value of ∼100 protein A molecules per spherule (Table 1) is near saturation for the spherule interior membrane surface area. We modeled 50 7-nm-diameter spheres representing protein A adjacent to the membrane surface within a tomographic model of half a typical spherule (Figure 7) to demonstrate how protein A may pack into the spherules.

The resulting near-full occupancy of the interior membrane surface area by protein A (Figure 7) and the nature of protein A as a transmembrane protein whose self-interaction is required for RNA replication [38,52] imply that the ∼100 copies of protein A form an inner network or shell within the spherule (Figure 7B). Such a shell would explain the formation and maintenance of the high-energy membrane deformation of spherules. A shell of these dimensions appears reasonable, given that the main shell of a reovirus core is 60 nm in diameter and is composed of 120 copies of a slightly larger protein λ1 (142 kDa) [59]. The distribution of FHV spherule size spans a defined range of ∼30–45-nm intramembrane diameter, suggesting some flexibility in the assembly of the protein A shell. Other examples of high-density protein shells of flexible size and shape include the capsids of retroviruses, influenza, and retrotransposons [60–62]. For example, one species of Ty retrotransposon forms virus-like capsids that have a 30–50-nm range of diameters, similar to FHV spherules, and contain on average 300 copies of a 381–amino acid protein subunit [62], a protein content very close to the FHV spherule average of 100 copies of 998–amino acid protein A. Endocytic vesicles, secretory transport vesicles, and synaptic vesicles are further examples of protein-induced membrane vesicles that each have a range of variable sizes (50–100-nm diameter), despite being formed by regular arrays of uniform proteins [63,64].

Because ∼90% of protein A, the FHV RNA polymerase (Figure 1), and ∼70% of newly synthesized FHV RNA (Figure 2) are spherule-associated, and essentially all FHV (−)RNA templates are membrane associated (P. Van Wynsberghe, P. Ahlquist, unpublished data), it appears likely that (−)RNAs, and thus any double-stranded RNAs (dsRNAs) are within spherules. Sequestration of dsRNA within such a compartment may allow the virus to avoid, minimize, or delay dsRNA-induced host–cell defense responses such as protein kinase, RNA activated (PKR) and RNase L [65] or RNA interference (RNAi) [66]. Such dsRNA localization is consistent with earlier observations of virus-induced membrane spherules containing fibrils with salt-dependent nuclease sensitivity [25,67].

The ∼100 protein A molecules per spherule (Table 1) would consume ∼18,300 nm^3^ of interior volume, leaving ∼14,000 nm^3^ within an average spherule to accommodate FHV RNA. Based on 0.655 nm^3^ per hydrated nucleotide for the crystal structure of duplex RNA [43,68,69], the volumes of FHV RNA1, RNA2, and RNA3 would be 2035, 917, and 254 nm^3^, respectively. Thus, in addition to ∼100 protein A molecules, a spherule of average size has enough interior space to contain at most four single-stranded RNA (ssRNA) or two dsRNA copies of all three FHV RNA species. Given this maximal occupancy, the estimate from biochemical data of an average of one (−)RNA1 and two (−)RNA2 templates per spherule (Table 1), together with at least one nascent (+)RNA progeny strand for each, appears fully reasonable.

Currently, it is not known if FHV RNA1 and RNA2 are replicated in separate or common spherules. If RNA1 and RNA2 were in separate spherules (i.e., 50% of spherules containing RNA1 and 50% containing RNA2), then the ratios of (−)RNA1 and (−)RNA2 to total spherules (Table 1) imply each RNA1-containing spherule would have two replication intermediates, and each RNA2-containing spherule would have approximately four replication intermediates. Because RNA1 (3.1 kb) is twice as long as RNA2 (1.4 kb), the total RNA content in both cases then would be nearly equal. If RNA1 and RNA2 were together in the same spherule, then each spherule would hold on average three replication intermediates (one RNA1 and two RNA2). The possibility of spherules containing both species of RNAs is intriguing, considering the interactions of FHV RNAs required for replication: FHV subgenomic RNA3, which is templated from RNA1, transactivates RNA2 replication and, in turn, RNA3 replication is suppressed by the resulting progeny RNA2 [70]. RNA3, and not its protein product, is responsible for transactivating RNA2 [70]. However, it is also possible that RNA3 is produced in one spherule during RNA1 replication and then exported to the cytoplasm prior to transactivating RNA2.

### Parallels with Other Viral RNA Replication Complexes

Membrane spherules similar to those of FHV are induced by many other (+)RNA viruses including alphaviruses [5], other members of the alphavirus-like superfamily [15], rubiviruses [7,20], flaviviruses [21], tombusviruses [22], and others [4,23–25]. Among these, one of the best-studied with regard to the localization and stoichiometry of RNA replication complex components is BMV. BMV and FHV differ in many important respects including that BMV encodes a much larger complement of RNA replication proteins [15,19]. Nevertheless, although the understanding that we present here for FHV RNA replication complexes is more advanced in many ways, the known characteristics of BMV RNA replication complexes are strikingly similar to those for FHV. Both BMV and FHV induce spherules of similar dimensions where viral RNA synthesis and viral replication proteins are localized [15]. BMV replication protein 1a, which is sufficient to induce spherules [15], is also a strongly membrane-associated [71], self-interacting [72,73] protein that is present at high copy number per spherule [15]. Similarly, whereas the ultrastructural organization of hepatitis C virus RNA replication complexes has not been defined, recent results suggest that these may also involve a dramatic excess of nonstructural protein copies per (−)RNA [74].

In addition to the many (+)RNA viruses whose RNA replication is associated with spherules, other (+)RNA viruses induce various, apparently distinct membrane rearrangements [4,17,21,26,27,75,76]. Although some or most of this variability reflects real ultrastructural differences, at least some of the perceived differences may be due to differences in perspective under conventional 2-D imaging. Our tomography results demonstrated that equivalent FHV spherules appeared to vary in morphology and topological relation to adjacent membranes when viewed in two dimensions from different perspectives (Figures 3C and 3D). A greater understanding of the 3-D nature of membrane rearrangements associated with RNA replication by other (+)RNA viruses may reveal shared features or common underlying principles.

Based on results with BMV, Schwartz et al. identified potential parallels between the assembly, structure, and function of membrane-associated RNA replication complexes and the cores of reverse-transcribing and dsRNA virus virions, including the sequestration of genomic RNA templates within a virus-induced compartment for replication [15,19]. The results presented here for FHV validate and extend these parallels by showing that all FHV spherules are membrane invaginations topologically equivalent to a budding, enveloped virion (Figure 4), and that self-interacting, transmembrane protein A is present at levels sufficient to coat the inner spherule membrane in a multi-subunit shell similar to the capsids of retrovirus and dsRNA virus cores (Figure 7). As with dsRNA viruses, hepadnaviruses, and retroviruses, the high copy of protein A per spherule suggests that there may be threshold effects in replication protein expression to initiate replication. Further analysis of the structure, interactions, and function of FHV RNA replication complexes should provide additional insights into the basic mechanisms of (+)RNA virus replication and potentially identify new approaches for antiviral interference.

## Materials and Methods

### Cells and infection protocol.

*Drosophila* S2 cells were grown at 28 °C in Gibco *Drosophila* serum-free media (SFM). Cells were dislodged by gentle scraping, pelleted, and resuspended at 10^7^ cells/ml. FHV was added at a multiplicity of infection of 10 for all experiments. The cells and virus were incubated at 26 °C on a rotary shaker at 1,000 revolutions per minute (rpm) for 1 h to let the virus attach. After the hour incubation, the cells were plated onto a tissue culture dish and further incubated at 28 °C.

### Mitochondria isolation.

Mitochondria were isolated from *Drosophila* cells as described by Echalier [77]. Briefly, cells were recovered by scraping and centrifugation and resuspended in a hypotonic buffer that contained 20 mM *N*-2-hydroxyethylpiperazine-*N'*-2-ethanesulfonic acid** (**HEPES; pH 7.4), 1 mM EGTA, and a protease inhibitor cocktail (1 mM phenylmethanesulphonylfluoride, 5 μg/ml pepstatin A, 1 μg/ml chymostatin, 10 mM benzamidine, 10 μg/ml leupeptin, and 0.5 μg/ml bestatin). After a 10 min incubation at room temperature, an equal volume of double isotonic buffer was added that consisted of the hypotonic buffer plus 0.5 M mannitol. The cells were lysed for 7 min using a pre-chilled Potter-Elvehjem homogenizer fitted with a Teflon pestle (Kimble-Kontes; http://www.kimble-kontes.com/) and attached to a stirrer motor spinning at 250 rpm. The lysate was transferred to a Dounce homogenizer fitted with a type B glass pestle (Kimble-Kontes) and disrupted manually for 100 strokes on ice. Unbroken cells and nuclei were removed by two 10 min centrifugation steps at 500*g* at 4 °C. Mitochondria were pelleted by centrifugation at 3700*g* for 10 min at 4 °C, resuspended in an isotonic buffer containing 0.25 M mannitol, and washed by a second centrifugation at 7000*g*. BrUTP incorporation on the isolated mitochondria was performed at 28 °C for 1 h as described previously [15].

### BrUTP transfection.

*Drosophila* cells were infected with FHV as above. At 8 hpi, cells were treated with 20 μg/ml actinomycin D for 30 min. FuGENE 6 (Roche; http://www.roche.com) was diluted 10-fold in phosphate buffered saline pH 7.4 and mixed with BrUTP and actinomycin D to final concentrations of 10 mM and 20 μg/ml, respectively. The FuGENE/BrUTP/actinomycin D mix was incubated for 15 min at room temperature then added to the cells and incubated at 4 °C for 15 min. After the 4 °C incubation, the cells were moved to 28 °C for a 15-min labeling period and then immediately fixed and processed for EM.

### Monoclonal antibody.

Mouse monoclonal antibodies against FHV protein A have been described previously [9]. MAb clone 2–1.1.2.4.8, which recognizes the protein A epitope between amino acids 230 and 399, was used for immunogold EM labeling.

### Immunogold EM labeling .

BrUTP immunolabeling fixation was performed as described previously [15], except that samples were embedded in LR Gold resin. Samples were sectioned and placed on nickel grids. Sections were blocked with a goat-blocking solution (Aurion; http://www.aurion.nl), and incubated for 1 h with an anti-BrU antibody (PRB-1; Molecular Probes; http://probes.invitrogen.com), diluted 1:100 in an incubation solution containing 100 mM phosphate-buffered saline pH 7.4 and 0.1% BSA-c (Aurion). Grids were washed six times in incubation solution without antibody, then incubated for 2 h with a goat-anti-mouse antibody conjugated to an ultrasmall gold particle (Aurion) that was diluted 1:100 in incubation solution, and washed six times again with incubation solution. Silver enhancement was performed for 30 min using R-GENT SE-EM (Aurion). Protein A immunogold EM was performed in the same manner using the mouse monoclonal antibody at a dilution of 1:100. Background labeling was determined using uninfected control cells. Labeling density was determined by calculating the surface area of spherules, mitochondria, and cytoplasm using the point-hit method [78]. Specific labeling was determined by subtracting the background labeling density.

### Northern blot analysis and quantitation of FHV RNA.

Northern blotting was done as described previously [8]. The number of molecules of FHV RNAs was determined by comparison with a serial dilution of a known amount of in vitro transcripts representing a known amount of (+)RNA or (−)RNA molecules. RNA levels were quantitated with ImageQuant software (Molecular Dynamics; http://www.mdyn.com/).

### Western blot analysis and quantitation of FHV protein A.

Western blotting was done as described previously [8]. The number of molecules of protein A was determined by comparison with a purified protein A standard. To generate the standard for quantitation, protein A was expressed in Escherichia coli as described previously [8]. To purify protein A, the hydrophobic transmembrane domain of protein A was deleted (amino acids 8–89), replaced with a C-terminal His_6_ tag, and purified by talon column (Clontech; http://www.clontech.com) affinity chromatography. To further purify protein A, we performed preparative electrophoresis using a BioRad mini-prep cell. A 6%, 9.5-cm gel was run at 200 V for 9 h with an elution speed of 150 μl/min. Fractions containing the purified, truncated protein A standard were collected and quantitated based on comparison with known standards of bovine serum albumin and β-galactosidase. We quantitated protein levels with Lumi-imager software (Roche).

### EM.

For conventional transmission EM, cells were fixed and embedded as previously described [8]. For electron tomography, cells were fixed in 2% parafomaldehyde and 2.5% glutaraldehyde in 0.1 M sodium cacodylate, pH 7.4, post-fixed in 1% osmium tetroxide with 0.8% potassium ferrocyanide in sodium cacodylate buffer, stained with 2% uranyl acetate, dehydrated in a graded series of ethanol, and embedded in Durcupan ACM resin.

To calculate the total number of FHV-induced mitochondrial spherules per cell, FHV-infected *Drosophila* S2 cells were collected at 12 hpi, processed for transmission EM, and sectioned into 70-nm-thick slices. For each of 25 randomly selected cells imaged in these sections, we then counted all observable spherules with diameters larger than 20 nm. The number of spherules counted for each cell then was divided by the relevant section volume, which was calculated by measuring the cell area using ImageJ (National Institutes of Health) and multiplying by the effective section thickness. The effective section thickness is a correction used to avoid overcounting spherules with centers outside of the 70-nm physical section, which would otherwise be counted twice if adjacent sections were analyzed. As previously used to calculate synaptic vesicles per cell [79], this effective section thickness is the thickness that would encompass the centers of all counted spherules. In this case, the effective section thickness was 116 nm, based on adding 23 nm (the distance from the spherule center to a radius-perpendicular plane bisecting the spherule to yield a 20-nm-diameter section) to each face of the 70-nm section. The number of spherules per cell was calculated by multiplying the resulting density of spherules by the average volume of the almost perfectly round, 10 μm-diameter *Drosophila* S2 cells [56] (and our independent, matching measurements).

### EMT.

Three separate FHV infections produced samples for six independent tomograms. To survey the preservation quality and FHV-infection efficiency of the *Drosophila* cells, thin-sectioned material (∼80 nm thick) was examined using a JEOL 1200FX electron microscope. 3-D reconstructions of portions of the cell containing FHV-infected mitochondria were generated using current techniques of electron tomography [80]. Sections were cut with a thickness of ∼250 nm from blocks exhibiting well-preserved ultrastructure. These sections were stained for 30 min in 2% aqueous uranyl acetate, followed by 30 min in lead salts. Fiducial cues consisting of 20-nm colloidal gold particles were deposited on both sides of the section. For each reconstruction, a series of images was collected with a JEOL 4000EX intermediate-voltage electron microscope operated at 400 kV. The specimens were irradiated before initiating a tilt series in order to limit anisotropic specimen thinning during image collection. Pre-irradiation in this manner subjected the specimen to the steepest portion of the nonlinear shrinkage profile before images were collected. Six tilt series were collected: five single-tilt and one double-tilt. “FHV2” was the highest resolution single-tilt reconstruction and “FHV6” was the high-resolution double-tilt reconstruction; the majority of the analyses were conducted on these two reconstructions. The single-tilt series were recorded at 40,000 magnification with an angular increment of 2 ° from −60 ° to +60 ° about an axis perpendicular to the optical axis of the microscope using a computer-controlled goniometer to increment accurately the angular steps. These single-axis tilt series were collected using a CCD camera with pixel dimensions 1,960 × 2,560. The pixel resolution was 0.55 nm. The illumination was held to near parallel beam conditions and optical density maintained constant by varying the exposure time. The IMOD package was used for generating the reconstructions [81].

Double-tilt tomography was performed by first collecting two tilt series of the same cellular region around orthogonal axes. After the first tilt series was complete using an angular increment of 2 ° from −66 ° to +66 °, the specimen grid was rotated 90 °, and the second tilt series was acquired from −60 ° to +66 °. The IMOD software suite was used for fiducial mark tracking and alignment. The positions of 30 gold particles were tracked in both tilt series. After alignment, the tomographic reconstruction was generated by a projective algorithm [82].

Volume segmentation was performed by manual tracing in the planes of highest resolution with the program Xvoxtrace [83]. The mitochondrial reconstructions were visualized using Analyze (Mayo Foundation, Rochester, MN, United States), ImageJ (National Institutes of Health), or the surface-rendering graphics of Synu (National Center for Microscopy and Imaging Research, San Diego, CA, United States) as described by Perkins et al. 2001 [84]. These programs allow one to step through slices of the reconstruction in any orientation and to track or model features of interest in three dimensions. Measurements of structural features were made from planes within the reconstructed volume with the program ImageJ (National Institutes of Health) or within segmented volumes by the programs Synuarea and Synuvolume (National Center for Microscopy and Imaging Research). Some 3-D maps, images, and videos were created using the software Amira (Mercury TGS; http://www.tgs.com).

## Supporting Information

Video S1 Animation through a *z*-Series of Slices of a Double-Tilt Tomogram Showing FHV-Induced Spherule Rearrangements of a Mitochondrion (8.1 MB MOV)

Video S2 *Z*-Series Animation Illustrating the Varied Appearance of Spherule Clusters when Sectioned Parallel and Perpendicular to the Axes through Spherule Necks Note how the morphology of the mitochondrion near the center changes during the progression through the *z*-series.
(1.3 MB MOV)

Video S3 Relationship of a 3-D Map of FHV-Induced Spherule Rearrangements of a Mitochondrion and the Electron Density from which the Map was Derived Blue indicates outer mitochondrial membrane, white indicates FHV spherules, yellow indicates inner mitochondrial membrane. The red arrow points along the *y*-axis and the green arrow points along the *x*-axis of the tomogram. The boundary of the total tomographic volume is outlined by the orange bounding box.
(5.8 MB MOV)

Video S4 A 90 ° Rotation of Figure 4B to Figure 4C (1.0 MB MOV)

## Abbreviations

(−)RNA: negative-strand RNA
(+)RNA: positive-strand RNA
BMV: brome mosaic virus
BrUTP: 5-bromouridine 5'-triphosphate
dsRNA: double-stranded RNA
EM: electron microscopy
EMT: electron microscope tomography
FHV: flock house virus
hpi: h post infection
